# Environmental Disinfection in Long-Term Care Facilities—A Scoping Review

**DOI:** 10.3390/microorganisms14071408

**Published:** 2026-06-26

**Authors:** Yinan He, Wing Sum Lo, Pak Leung Yuen, Patricia Tai Yin Ching, Eric Po Tung Sze, Kin On Kwok, Margaret Ip, Christopher Koon Chi Lai

**Affiliations:** 1 Department of Microbiology, Faculty of Medicine, The Chinese University of Hong Kong, Lui Che Woo Clinical Sciences Building, Prince of Wales Hospital, Shatin, New Territories, Hong Kong SAR, China; yinanhe@cuhk.edu.hk (Y.H.); margaretip@cuhk.edu.h (M.I.); 2 Faculty of Medicine, The Chinese University of Hong Kong, Hong Kong SAR, China; 3 Hong Kong Branch, The Institute of Healthcare Engineering and Estate Management, Hong Kong SAR, China; ccpe@netvigator.com; 4 WHO Collaboration Centre for Infectious Disease Epidemiology and Control, School of Public Health, The University of Hong Kong, Hong Kong SAR, China; chingpty@yahoo.com.hk; 5 Department of Chemistry, The Chinese University of Hong Kong, Hong Kong SAR, China; tpesze@cuhk.edu.hk; 6 Jockey Club School of Public Health and Primary Care, The Chinese University of Hong Kong, Hong Kong SAR, China; kkokwok@cuhk.edu.hk

**Keywords:** environmental disinfection, long-term care facility, nursing home, no-touch technology, infection control

## Abstract

**Background:** Long-term care facility (LTCF) residents are highly susceptible to healthcare-associated infections, and prevention is challenging given frailty, dementia, communal living, and resource constraints. Environmental surface and air contamination contribute to transmission. Novel no-touch automated disinfection technologies have been studied in hospitals, but evidence specific to LTCFs is scarce. This scoping review summarizes recent LTCF-focused interventions, their effectiveness, and implementation considerations. **Methods:** This scoping review was conducted following the Preferred Reporting Items for Systematic reviews and Meta-Analyses extension for Scoping Reviews (PRISMA-ScR) Checklist. We searched PubMed, Medline, Embase, CINAHL, and Scopus for observational or experimental studies evaluating environmental disinfection in LTCFs/nursing homes, excluding body decolonization, non-LTCF settings, and reviews/protocols. Two reviewers independently screened and extracted data via Covidence. This review has been registered on OSF (Open Science Framework). **Results:** Of 1491 records, 7 studies met the inclusion criteria (6 from the USA, 1 from Australia): one cluster randomized trial, one interrupted time series studies, three prospective observational studies, and two pre–post designs. Interventions included physical methods (HVAC-integrated UV/UVGI, continuous UVGI) and chemical approaches (dry hydrogen peroxide, room fogging plus chlorine dioxide wipes, hydrogen peroxide wipes). Outcomes were heterogeneous (surface SARS-CoV-2 RNA, COVID-19 attack/case rates, airborne/surface microbial loads, and one clinical endpoint—acute respiratory illness). Several studies reported reductions in environmental or airborne bioburden; however, UV-based studies did not demonstrate statistically significant reductions in clinical infections. Certainty was limited by small numbers, non-randomized designs, and diverse outcome measures. **Conclusions:** No-touch automated disinfection methods appear promising as supplements to standard infection prevention control bundles for reducing environmental contamination in LTCFs. Nevertheless, consistent clinical benefits are unproven. Rigorous, LTCF-tailored, adequately powered trials with standardized clinical and environmental outcomes, plus implementation and cost-effectiveness evaluations, are needed.

## 1. Introduction

Residents in long-term care facilities (LTCFs) often experience disproportionately high infection and mortality rates from acute respiratory tract infections and multiple drug-resistant organisms [1,2,3,4]. The implementation of infection prevention and control (IPC) measures are uniquely challenging in the LTCF setting owing to the population’s physical condition, the facility’s residential nature, resource constraints [5,6,7,8], lower staffing ratio than hospitals, and compromised hand hygiene compliance due to time pressure [9,10,11,12]. Standard infection control interventions are more difficult to implement in LTCFs than in hospital settings as dementia and frailty can impair adherence to handwashing and physical distancing recommendations. Physical distancing can have detrimental effects on residents as it can exacerbate residents’ susceptibility to loneliness and mental illness and is thus challenging to implement [13,14]. The advanced age of LTCF residents is also associated with a higher rate of comorbidities that place them at increased risk for infection and mortality [6,8].

Substantial scientific evidence has accumulated that contamination of environmental surfaces in hospital rooms plays an important role in the transmission of several key healthcare pathogens, including methicillin-resistant *Staphylococcus aureus* (MRSA), *vancomycin-resistant Enterococci* (VRE), *Clostridioides difficile*, *Acinetobacter* spp., *Pseudomonas aeruginosa*, norovirus, and *Candida auris* [12,15,16]. Routine manual cleaning with low-level chemical disinfectants has been regarded as the first line of defense against the spread of pathogens [17,18,19]. They are highly effective in eliminating pathogens from environmental surfaces, often achieving reductions of approximately 3 log10 (≥99.9% reduction in viable counts). Consequently, rigorous daily cleaning of high-touch surfaces (e.g., bedrails, doorknobs) in LTCFs to prevent healthcare-associated infections (HAIs) has been strongly emphasized [20]. However, multiple studies have demonstrated that despite terminal cleaning and disinfection with these low-level chemical disinfectants, room surfaces are frequently contaminated with multidrug-resistant pathogens, due to incomplete surface cleaning and disinfection of hard contact surfaces [21,22]. For the purposes of this review, we use the term ‘terminal cleaning/disinfection’ to refer to whole-room cleaning and disinfection conducted after a resident or patient leaves the room or at the end of an occupancy period, with the goal of reducing environmental contamination to very low or undetectable levels before the next admission. Recent studies have further shown minimal beneficial effects from standard IPC interventions in LTCFs due to high staff–resident ratios, limited supplies, and communal living [4,23].

Several novel no-touch automated technologies for environmental disinfection, including methods for terminal disinfection, continuous room disinfection, and coated self-disinfecting surfaces, have been studied in hospital settings [16,24]. For instance, it was proven that ultraviolet-C (UV-C) light systems achieve at least a 99% pathogen reduction in healthcare facilities [25]. The increased application of hydrogen peroxide vapor (HPV) for terminal disinfection in hospitals has been proven to be efficacious against a wide range of microorganisms, environmentally friendly, and easy to use [26]. In addition, self-disinfecting surfaces are an emerging area in the field of decontamination in healthcare facilities, involving coatings with heavy metals that enable them to interact with microorganisms to achieve infection prevention [27]. However, most studies are in hospital or laboratory settings, and few studies have examined their use in LTCF settings.

HCAIs are a major cause of morbidity and mortality among residents of LTCFs [28]. The US Centers for Disease Control and Prevention (CDC) estimates that 1 to 3 million serious infections occur in LTCF settings every year [29]. Together with the context of an aging global population [30], the need for better methods to improve the living environment in LTCFs is of ever-increasing importance. Therefore, this scoping review aims to provide an update to the full range of the latest disinfection interventions studied in LTCFs, identify and map the available evidence regarding efficacy and potential implementation barriers in LTCFs, and reveal gaps in the research literature to guide future primary studies.

## 2. Materials and Methods

This scoping review was conducted following the Preferred Reporting Items for Systematic reviews and Meta-Analyses extension for Scoping Reviews (PRISMA-ScR) Checklist (Table S1), and guided by the Population–Concept–Context (PCC) framework to define the scope and inclusion criteria. Population: Residents and staff in long-term care facilities (LTCFs) or nursing homes, particularly those vulnerable to healthcare-associated infections due to frailty, dementia, communal living, and resource constraints. Concept: Environmental disinfection interventions, including novel no-touch automated technologies (such as UV-C/UVGI, hydrogen peroxide vapor/fogging, chlorine dioxide wipes) and chemical/physical methods aimed at reducing surface and air contamination and pathogen transmission. Context: Long-term care facilities and nursing homes (excluding hospitals, restaurants, or other public settings); studies published between 1 January 2021 and 2 May 2026; observational or experimental designs reporting efficacy outcomes (such as microbial reduction, pathogen RNA detection, and infection rates). This scoping review has been registered on OSF (Open Science Framework), and is available at https://osf.io/hdkzs (Accessed 12 April 2025).

### 2.1. Inclusion and Exclusion Criteria

There was no language limitation in this review. Titles and abstracts published in languages other than English were screened using online translation tools or team members with relevant language proficiency, and full texts were translated as needed for eligibility assessment and data extraction. Observational and experimental studies published between 1 January 2021 and 2 May 2026 on the effect of environmental disinfection carried out in long-term care facilities or nursing homes were included. Exclusion criteria included (1) studies that were not related to environmental disinfection, but other related topics, such as decolonization on the body, antibiotic treatment, or disinfection of reusable medical materials; (2) studies that were not conducted in LTCFs, but in hospitals, restaurants, or other public gathering locations; (3) studies that failed to report at least one outcome measure to reflect the efficacy of the approach applied for environmental disinfection; (4) review and protocol studies.

### 2.2. Study Search and Selection

Multiple databases—PubMed, Medline, Embase, CINAHL, and Scopus—were searched for potential studies published between 1 January 2021 and 2 Mayy 2026. The detailed searching strategies were shown in Table S2. Two reviewers independently screened articles via the Covidence system.

### 2.3. Data Extraction and Quality Assessment

A standardized data extraction form was developed to systematically retrieve relevant information from the included studies. For each eligible article, the following data items were extracted: author(s) and publication year; country where the study was conducted; study design (e.g., observational studies, randomized clinical trial, or other designs); approach or interventions for disinfection (e.g., chemical agents, ultraviolet light, dry hydrogen peroxide, or other methods); setting (e.g., specific nursing home units such as long-term care wards, skilled nursing facilities, or assisted living sections); and effect on clinical infection/outcomes (e.g., changes in healthcare-associated infection rates, reduction in environmental bioburden, or microbial colonization on surfaces). Data extraction was performed independently by two reviewers, and disagreements were resolved through discussion or consultation with a third reviewer.

As this review follows the scoping review methodology, a formal quality assessment of the included primary studies was not conducted. Consistent with the aims of scoping reviews that focus on mapping the available evidence, identifying knowledge gaps, and characterizing the breadth of the literature rather than critically evaluating the internal validity or synthesizing effect estimates, we did not apply quality appraisal tools.

## 3. Results

### 3.1. Study Selection and Characteristics

Initially, a total of 505 studies were found in PubMed, 86 articles were matched in Medline, 242 studies were obtained from Embase, 198 articles were identified in CINAHL, and Scopus contibuted 460 articles. After removal of 206 duplicated records, 1285 studies were retained to be screened using their title and abstract. During this process, 1224 irrelevant articles that mainly focused on topics of decolonization on the body, treatment with antibiotics, or disinfection of reusable medical materials were excluded, and 42 articles that were review studies, protocols, or letters to the editor were also removed, leading to 19 articles to be subjected to the full-text screening. Subsequently, 12 studies were rejected for the following reasons: 3 studies were not conducted in LTCFs but in acute-care facilities, hospitals, or clinics; 4 studies failed to report relevant effects or outcome measures of the disinfection method on the environment; 3 articles were laboratory experiments without involving a population; and 2 articles only published the abstract with the full text missing. Ultimately, seven studies were included in this review (Figure S1).

Table 1 shows the characteristics of the seven included studies. Among the seven selected studies, except for one study conducted in Australia [31], all were carried out in the USA [32,33,34,35,36,37]. The study designs were heterogeneous, with two pre–post studies, three prospective observational studies, one interrupted time series, and one cluster randomized clinical trial design [31,32,33,34,35,36,37].

The outcome measures reflecting the effect of environmental disinfection varied among the seven studies. Three studies applied COVID-19-related outcomes, and among them, one adopted the SARS-CoV-2 RNA detection rate on surfaces of LTCFs [32], one made use of the COVID-19 attack rate [33], while another directly used the number of COVID-19 cases [34]. Two studies applied microbial reduction [35] and the airborne pathogen reduction rate [36], and another study employed the colonization detection rate on surfaces [37]. Only one study utilized an outcome measure at the clinical outcome level, using the incidence rate of acute respiratory illness (ARI) as the endpoint. In terms of methods used for environmental disinfection, three studies applied physical methods, while four studies deployed chemical disinfectants. Of the three articles that studied physical disinfection methods, two incorporated ultraviolet (UV) light into the HVAC system, and one utilized a continuous UV germicidal irradiation appliance. The other four studies involving chemical methods included two using dry hydrogen peroxide, one using room chemical fogging and chlorine dioxide-based wipes, and one using hydrogen peroxide wipes.

### 3.2. Effect of Physical Disinfection Method

Shoubridge et al. conducted a multicenter, two-arm, double-crossover, cluster randomized clinical trial to determine the effect of germicidal UV, or UV germicidal irradiation (UVGI) appliances on ARI incidence in Australia [31]. The four LTCFs were divided into two equal-sized zones, with 44 beds per zone. In each arm, zones were randomly allocated active UVGI appliances as the intervention or inactive appliances as the control. They reported that the overall difference in the number of infections per zone between the intervention and control was −0.36 (95% CI, −1.09 to 0.37), though the difference was not statistically significant.

Jutkowitz et al. introduced an ultraviolet air purification system installed in the existing air condition system in 81 nursing homes [34]. They compared COVID-19 infection and death cases before and after installation of the system. After installation, there were 1.69 (95% CI, −4.32 to 0.95) fewer COVID-19 cases per 1000 residents per week, but such reduction was not statistically significant. Regarding the mortality rates, the results showed no difference between the two periods.

Urrutia et al. confirmed the beneficial effect of an advanced air purification technology (AAPT) installed in air conditioning systems [36]. The AAPT was a heat-ventilation air conditioning system which combined proprietary molecular media for volatile organic compound (VOC) removal, high-dose ultraviolet germicidal irradiation (UVGI) for single-pass pathogen inactivation, and high-efficiency particulate air (HEPA) filtration. Compared with the control floor, on which only a HEPA filter was applied, the intervention floor (with AAPT) showed a 98.83% decrease in airborne pathogens, an 89.88% reduction in VOC load, and a significant decrease in surface pathogens in most sampled locations, concluding that the comprehensive air purification significantly reduced both airborne and surface microbial contamination.

### 3.3. Effect of Chemical Disinfection Methods

The effectiveness of using hydrogen peroxide for environmental disinfection was investigated in three studies. While one examined the use of hydrogen peroxide wipes, the other two explored the effect of dry hydrogen peroxide (DHP). In 2023, Cole et al. [35] evaluated the impact of dry hydrogen peroxide (DHP) devices deployed in a 15-bed neurobehavioral unit of an LTCF, with the reduction in surface microbial load and VOC levels as the outcome measures. During the study, 19 standalone DHP units were installed in patient rooms and communal areas as the intervention, which operated continuously to generate DHP from ambient air. The study concluded that DHP can significantly reduce the environmental bioburden in occupied LTCF settings, with average colony-forming units decreasing by 61.6% on day 14, 86.6% on day 28, and 89.8% on day 55 compared to the baseline. On the other hand, the VOC levels were found to be significantly decreased by exposure to DHP.

The same group examined the use of DHP in LTCF further in another study in 2025 [33], and studied the clinical infection rates during the COVID-19 outbreak as the outcome. The study was carried out across three units in an LTCF: two units had DHP deployed, while one did not. The COVID-19 attack rate differed markedly among the units. The unit without DHP experienced an attack rate of 66.67%, while the two DHP-equipped units either suffered a lower attack rate of 13.33% or no occurrence of COVID-19 cases.

Lesho et al. evaluated the comparative efficacy of different environmental cleaning and disinfection strategies in an acute-care hospital and an LTCF during COVID-19-related staff shortages. The strategies included daily cleaning, enhanced terminal cleaning (involving wet wiping with a sporicidal disinfectant), and contingency-based professional remediation (primarily chemical fogging). The reduction in SARS-CoV-2 RNA detected on high-touch surfaces was measured to assess cleaning effectiveness. Key findings in the LTCF indicated that contingency cleaning relying solely on chemical fogging without manual wiping was substantially less effective. In contrast, room fogging combined with wiping using a proprietary chlorine dioxide-based disinfectant resulted in an 11% reduction in the proportion of positive surfaces [32].

Sansom et al. performed a prospective multicenter observational trial and studied the environment contamination detection and recontamination rate in six ventilator-capable skilled nursing facilities. *C. auris* contamination was detected on 32.2% (66/205) of room surfaces before disinfection and 20.5% (39/190) of room surfaces by 4 h after disinfection [37].

## 4. Discussion

We performed a scoping review to map the breadth and characteristics of existing research in this area, as the available evidence is limited (*n* = 7) and exhibits substantial heterogeneity in study design and reported endpoints. This approach allowed for an overview, the identification of key concepts and gaps, and an assessment of the feasibility of future synthesis. We believe a systematic review that attempts quantitative synthesis would be unreliable given the small and diverse evidence base. This review collectively highlights a evidence base that is growing, albeit slowly, for the use of the latest chemical and physical environmental disinfection technologies in LTCFs. Similar reviews have been performed but in different settings (hospitals, settings with children, or in laboratory environments), have studied prevention bundled approaches (with education, administrative measures, or other infection control measures such as hand hygiene), or have focused on outbreaks only [16,38,39]. To our knowledge, this review is the first to gather a variety of novel technologies for environmental disinfection in LTCFs. The findings imply a pattern shifting away from reliance on manual cleaning alone and toward the adoption of supplemental, automated, or continuous disinfection strategies to address persistent environmental contamination.

### 4.1. Current State of Environmental Disinfection in LTCFs

The inanimate environment in healthcare settings is a well-established reservoir for pathogens capable of causing infections, even if it is visually clean [40]. Pathogens can persist on environmental surfaces for hours to months, which enables them to be transmitted from contaminated surfaces, such as bed rails, door handles, call buttons, communal dining tables, and medical equipment, to the skin of residents or medical staff, acting as a secondary source even when direct person-to-person contact is prevented [41].

Routine cleaning and disinfection are often inadequate in LTCF settings, as evidenced by a recent study which found that over 90% of the sampled surfaces in 11 LTCFs had either failing adenosine triphosphate (ATP) scores or tested positive for a DNA bacteriophage found in the human gut [42]. This is consistent with the observations in the reviewed studies that demonstrated routine manual cleaning in LTCFs was often suboptimal and inconsistent, which are often exacerbated by staffing shortages, high turnover, and the complex care needs of residents [32,35]. These phenomena have also been reported in other studies, with inconsistent cleaning policies, variable staff training and wide interfacility variation in nursing home cleaning, and high turnover in these settings linking them to deficiencies in infection control [9,43]. Furthermore, outbreaks have been linked to breaches in the cleaning and disinfection of equipment and the environment in LTCF settings [44,45].

Automated tools have been adopted in hospitals to monitor cleaning performance. These include UV-C disinfection robots that can record room-entry and cycle completion data with digital auditing systems that can track whether specific high-touch surfaces have been cleaned, and video-or-sensor feedback platforms that provide notifications to environmental services staff when cleaning is incomplete or deviates from protocol [46,47]. Studies in acute-care settings suggest that such technologies can improve cleaning fidelity and reduce the proportion of missed high-touch surfaces, and in some cases, even achieve lower environmental contamination when combined with standard training and supervision [48,49].

However, implementation in LTCFs raises additional concerns including privacy and acceptability concerns around video monitoring in a residential environment, limited on-site technical support for complex devices and resource constraints that may limit widespread deployment of robotic platforms [50]. Future LTCF-specific studies should therefore not only evaluate the impact of such monitoring technologies on clinical outcomes but also examine resident and staff acceptance and cost-effectiveness in this distinct care context.

Traditional disinfection methods rely on intermittent manual cleaning with low-level disinfectants. However, a recent study showed that time to contamination was rapid, with 20.5% of room surfaces contaminated with MDROs including *C. auris*, MRSA, VRE and ESBL by 4 h after disinfection. The proportion of contamination remained stable between 4 and 12 h after disinfection [37]. These findings have spurred interest in ‘no-touch’ and automated technologies that can operate independently of direct staff intervention. No-touch automated methods are in demand as there is often a shortage of environmental service workers, and concerns about pathogenic organisms further complicate cleaning and disinfection efforts [39]. Such technologies have additional effects beyond their efficacy—implementing visible technologies has been shown to improve safety climates in hospitals by increasing the confidence of both patients and healthcare workers [51].

### 4.2. General Overview of No-Touch Environmental Disinfection Methods

Novel environment disinfection technologies that have been developed are ‘no-touch’ or automated based on the rationale discussed above. Apart from classifying them by their properties (chemical versus physical), these technologies can also be classified according to their usage: terminal disinfection, continuous room disinfection, and coating with self-disinfection materials [16] (Table 2).

Terminal disinfection technologies are used for entire rooms with all inhabitants evacuated. Commonly implicated devices include stationary UV devices (UV-C, UV-pulsed Zenon), or mobile devices (UV-C). Hydrogen peroxide systems utilize different concentrations of hydrogen peroxide vapor in 30–35% H_2_O_2_ or in 5–6% H_2_O_2_ and silver. There are also various hand-held UV devices and electrostatic sprayers to augment terminal disinfection. Continuous room disinfection technologies are designed to be used with chemical or physical agents that are considered safe to use without the need for room evacuation: chemical agents used include diluted hydrogen peroxide, hydroxyl radicals, and free reactive oxygen. Physical methods include far-UV (200–230 nm), UV-A (365 nm), and visible light (400–470 nm), and are commonly implicated for continuous room disinfection. These are ‘safer’ alternatives to UV-C, but it must be noted that they are not yet universally deemed safe for continuous, unrestricted exposure in occupied environments. For example, chronic exposure to UV-A can contribute to photoaging and increase skin cancer risk, and it can harm ocular tissues with sufficient or improper exposure. Their use should follow current regulatory guidance, device manufacturer specifications, and risk assessments. Self-disinfecting coating materials were studied and included heavy metals (e.g., copper, silver), quaternary ammonium compound-based agents, cationic polymers, photoactivated surfaces (e.g., TiO_2_), and antimicrobial peptides [52,53,54,55,56,57,58,59]. However, it must be emphasized that these no-touch disinfection methods should be considered as a supplement to manual decontamination and should not be considered as a replacement for manual cleaning [60].

### 4.3. Chemical Disinfection Methods in LTCF

In LTCFs, dry hydrogen peroxide (DHP) methods utilize oxygen and humidity from ambient air to generate dry hydrogen peroxide that reduces the presence of bacteria, viruses, and fungi, as well as volatile organic compounds, in occupied settings [61,62,63,64,65]. One study demonstrated a statistically significant 44.3% reduction in the incidence of hospital-acquired infections, including a 76.4% reduction in the incidence of *Clostridioides difficile* infection [62]. The findings of the two studies in our analysis suggest that DHP systems show promise for continuous microbial reduction on surfaces and in the air, correlating with significant reductions in bioburden and potentially lower infection attack rates [33,35].

### 4.4. Chemical Disinfection Methods in Other Settings

A hydrogen peroxide-based disinfection system that has been extensively studied in the hospital setting is the use of hydrogen peroxide vapor (HPV) for terminal disinfection [16]. Although most studies had relatively weak designs and confounders, such as hand hygiene compliance, environmental cleaning practices and frequencies, HPV was generally effective in reducing all MDROs, including CDI, VRE, MRSA, and ESBL-GNB. HPV systems had the advantages of being effective in all contiguous spaces and achieving high microbial inactivation but had a major disadvantage: they can only be used for terminal disinfection, requiring rooms to be devoid of people and all vents/ducts to be sealed.

### 4.5. Physical Disinfection Methods in LTCF

The three studies included in our analysis used continuous UV air purification with shielded UVGI integrated into HVAC systems. Its impact on airborne pathogen load and respiratory infection incidence was evaluated [31,34,36] and showed a modest reduction in airborne pathogen and VOC levels, an apparent decrease in the number of ARIs and HCAIs, and a reduction in attack rates in outbreaks. Continuous UVGI, the approach adopted in the three included studies, exerts air purification activity when air passes through a disinfection zone, shielded from zones of human activities, as a consequence of passive or fan-assisted air circulation. It must be emphasized that the irradiation time in these air purification systems is less than a few seconds, which may be insufficient for disinfection, and that the effect observed in these studies is due to the combined effect of the entire HVAC system or appliance, which is combined with HEPA filtration rather than UVGI alone.

### 4.6. Physical Disinfection Methods in Other Settings

Other physical disinfection methods extensively studied in hospital settings included UV-C and UV-PX in terminal disinfection. The majority of these studies demonstrate good efficacy, showing a reduction in CDI, MRSA, VRE, and drug-resistant Acinetobacter [16,66]. Limitations of the studies include that the study designs were generally weak, with the majority being before–after studies, retrospective cohorts, and quasi-experimental designs (non-randomized before–after or interrupted time series studies in which an intervention is introduced and outcomes are compared over time without random allocation). There is also failure to assess hand hygiene compliance, failure to assess cleaning compliance, and a lack of sample size calculations for negative studies. One major advantage of UV-C technologies is that it is unlikely that MDROs will become UV-resistant even after serial exposure [67].

Increased rates of air exchange in HVAC can reduce the risk of airborne viral transmission but are associated with considerable heating and cooling costs [68]. UV-C is harmful to humans and must be used in a way that avoids direct human contact and UV-C devices for terminal disinfection must have the room evacuated before use. However, UV-C can be used in rooms with occupants when shielded against direct human exposure. Continuous UVGI has also been shown to reduce airborne bacteria and may also lower the number of HAIs, including those caused by contact pathogens in hospital settings, resulting in lessened morbidity and lower costs. It has been suggested that healthcare facilities could consider continuous shielded UV-C at the room level as a possible addition to their infection prevention and control protocols [69].

Other physical methods have been studied: high-intensity visible violet light with a peak output of 405 nm (violet-blue light) was shown to kill epidemiologically important pathogens, and lower *C. difficile* spores after 72 h of exposure. Whether the level of these reductions is sufficient to reduce healthcare-associated infections remains uncertain, and this question requires further study [70]. A study using a benchtop device with a diffused array of light-emitting diodes (LEDs) producing UV-A light at 365 nm wavelength resulted in a modest reduction (1–2 log) in the recovery of MRSA, *Candida auris*, and bacteriophages, but not *C. difficile* spores after 24 h of exposure. When UV-A was used in a ceiling light fixture, this resulted in a significant reduction in the frequency of recovery of pathogenic microorganisms from in-use medical equipment. These findings suggest that UV-A could be useful as a means to provide continuous low-level decontamination of surfaces in healthcare facilities [71]. Far-UV-C light (222 nm) is another promising technology that may well prove to be effective at treating air and surfaces, without some of the safety precautions required for standard UVGI. Ceiling- and wall-mounted devices have been evaluated with moderate (1–4 log) reductions in vegetative bacteria and bacteriophages, but are less effective in reducing *C. auris* and *C. difficile* spores [71,72,73]. Far-UV-C light was also shown to kill influenza virus and SARS-CoV-2 [68,74,75]. It can be used in a full-sized room, even with continuous introduction of microbes into the space [76].

### 4.7. Choice of Study Endpoints

The endpoints of environment disinfection studies can generally be classified into four major groups: (1) process measures (cleaning frequency/fidelity), (2) environmental proxy outcomes (total microbial colony counts/viral RNA detection rates/VOCs), (3) intermediate transmission outcomes (colonization/recontamination), and (4) resident-centered clinical outcomes (ARI/HAI incidence, hospitalization, mortality).

In the seven LTCF studies, the reported endpoints were “environmental proxy outcomes,” such as surface contamination (e.g., SARS-CoV-2 RNA detection rates or total microbial colony counts) and air quality metrics (airborne pathogen counts or volatile organic compound levels), and “resident-centered clinical outcomes,” such as acute respiratory infection incidence, COVID-19 attack or incidence rates (including mortality), and general healthcare-associated infection (HAI) incidence. One intervention using advanced air filtration achieved a ~98.8% reduction in airborne pathogens and an ~89.9% reduction in VOCs, with a corresponding 39.6% drop in overall HAI rates [36]. Continuous dry hydrogen peroxide similarly reduced the surface bioburden in an outbreak investigation, where the COVID-19 attack rate was 0% in a treated unit versus 66.7% in an untreated unit [33]. Surface swabs for SARS-CoV-2 RNA have also been used (many surfaces have tested RNA-positive during LTCF outbreaks) [32], but it should be noted that PCR detection does not prove the presence of infectious viruses.

It must be emphasized that the endpoints in the selected studies are highly heterogeneous and often surrogate. These measures are useful for indicating environmental hygiene performance, but they do not automatically translate into reduced transmission or fewer infections. For instance, in Cole’s study [35], CFU-based (colony-forming unit) results depend strongly on sampling methods and recovery efficiency. A reduction in CFU indicates cleaner surfaces but not necessarily a lower risk of clinical infection, making cross-study comparisons difficult unless protocols are standardized. Another study chose volatile organic compound reduction as the endpoint [36]. While decreased VOCs may reflect improved air quality, is not a validated proxy for respiratory infection risk. For SARS-CoV-2 detection [32], PCR positivity on surfaces indicates the detection of viral RNA fragments, and multiple studies report that infectious virus is rarely recoverable from contaminated surfaces. RNA detection should be interpreted as evidence of contamination rather than proof of viable viruses or transmission risk. Although such surrogate endpoints suggest potential benefits for environmental hygiene, they disqualify a study from providing direct evidence of infection prevention, to some extent.

Importantly, most of the studies were underpowered for clinical endpoints. Thus, while heterogenous environmental endpoints demonstrate that novel disinfection technologies can reduce contamination, robust evidence linking these surrogate measures to fewer infections or deaths remains scarce [32,33,34], highlighting a clear evidence gap for future work.

### 4.8. Disparity with Hospital-Based Disinfection

Since 2015, there has been an exponential growth in good-quality studies that tie improvements in healthcare environmental hygiene to a reduction in healthcare-associated infections [77]. The Clean Hospitals Approach (the Clean Hospitals initiative), launched at the 2018 Healthcare Cleaning Forum, has grown into a collaborative public–private partnership with the common goals of increased communication, improved products and practices, and better patient safety [78]. In acute-care hospitals, the importance of environmental hygiene has been synthesized in several high-quality reviews. Thomas et al. [79] systematically reviewed the impact of cleaning and disinfecting surfaces in hospitals on reducing bacterial and viral infections. The study concluded that regular and thorough surface cleaning and disinfection significantly reduced the rates of hospital-acquired infections and proper disinfection protocols served as a critical measure to break the chain of pathogen transmission on environmental surfaces, helping control outbreaks of bacterial and viral infections in these settings. Another study from the same team focused on environmental service workers, and emphasized that investment in training, clear protocols, and regular assessment of cleaning activities enhanced the ability to reduce healthcare-associated infections through better environmental hygiene [80]. Similarly, Chau et al. reported in a systematic review and meta-analysis that hospital environmental cleaning bundles incorporating sporicidal agents and improved cleaning protocols were associated with significant reductions in *Clostridioides difficile* infection [81]. These hospital-based data provide strong proof-of-concept that optimizing environmental cleaning can yield clinical benefits, but the bundled nature of the interventions and the acute-care context limit direct extrapolation to LTCFs.

The contamination rate in LTCFs is not different from that in acute-care hospitals [32]. The use of novel disinfection methods is an attractive solution and has been examined in multiple studies in hospital settings [16]. In acute hospitals and operating theaters, environmental disinfection strategies often involve high-efficiency mechanical ventilation, HEPA filtration, and ultraviolet germicidal irradiation in some settings. Terminal disinfection technologies like UV-C devices and hydrogen peroxide vapor room decontamination are also used for operating theaters and high-risk wards. These involve rooms that are vacated and sealed between cases. Hospital studies have demonstrated that these technologies allow for substantial reductions in environmental contamination and decreases in infections like *C. difficile*, MRSA, VRE, and other multidrug-resistant organisms [82].

However, the findings cannot be directly applied to LTCFs due to distinct challenges in these environments when compared with acute hospital settings (Table 3). First, many LTCFs operate with limited financial resources, staff shortages, and high staff turnover, impacting the training and consistency of environmental services personnel [9,10]. In addition, infrastructure in LTCFs is varied in design, types of surface, and furnishings compared to hospitals; the environment also needs to maintain a homelike atmosphere for residents at the same time, which can affect cleaning efficacy and the suitability of certain disinfection technologies. In addition, the physical and social environment vary. LTCFs are primarily residential, where residents often spend considerable time in communal areas, such as dining rooms and lounges, and own many personal items, making terminal cleaning more complicated than in hospital wards, which usually have standardized patient rooms and can be emptied for disinfection [32,37]. Finally, LTCF residents are often at an advanced age, with cognitive impairments (such as dementia), and comorbidities leading to incontinence and functional dependence, which all lead to an increased risk of MDRO colonization and healthcare-associated infections and complicate control measures [2,35,37,83,84].

### 4.9. Persistent Challenges and Research Gaps

Despite the fact that technology is advancing, significant challenges remain. Evidence for direct infection reduction is still evolving. On the one hand, while encouraging outcomes in attack rates were observed in studies by Cole [33] and Shoubridge et al. [31], most research presented their results in environmental bioburden (CFU, RNA) or VOC levels, leading to the conclusion that robust, randomized controlled trials with clinical infection endpoints in LTCFs are relatively scarce. On the other hand, rapid recontamination is a critical issue, as evidenced by Sansom et al. [37], where surfaces near colonized patients were recontaminated with *C. auris* and MDROs within hours of disinfection, highlighting the limits of intermittent cleaning and the potential demand for long-acting disinfectants or antimicrobial surfaces.

Despite all the advantages of non-touch technologies, it must be emphasized that effective implementation to ensure devices are used and used correctly requires ongoing monitoring and feedback. Substantial time may be invested in training, monitoring usage, and locating devices and moving them from ward to ward. Many LTCFs operate under tight budgetary constraints, making the capital and maintenance costs of advanced HVAC upgrades, UVGI systems, or DHP units a major barrier, particularly in smaller or older facilities. High staff turnover and limited training time may impede consistent device operation and integration with existing cleaning workflows, while homelike design considerations (e.g., soft furnishings, personal belongings, communal lounges) can reduce the effectiveness of technologies that rely on line-of-sight or uncluttered surfaces. Residents with frailty or dementia may also be sensitive to noise, light, or visible equipment, affecting acceptability. Future primary studies in LTCFs should therefore incorporate implementation science frameworks, explicitly evaluating staff and resident acceptance, workflow integration, safety, and cost-effectiveness alongside microbiological and clinical endpoints [50]. Residual contamination of surfaces is also not uncommon after the use of ‘no-touch’ devices, particularly UV technologies [16]. It must also be emphasized that these advanced technologies are intended as an adjunct to, not a replacement for, manual cleaning. All guidance and studies emphasize that surfaces should be cleaned of dirt/organic matter first so that UV-C light can reach microbes. Casini et al. noted that UV-C treatment “has to be applied after cleaning because organic material could interfere with the effectiveness of the disinfection treatment” [46]. Knobling et al. deliberately omitted prior cleaning in their trial in order to test UV-C alone [85].

Patient privacy curtains are commonly used in long-term care facilities and nursing homes/old-age homes. They provide visual privacy for personal care (bathing, dressing, toileting), modesty during medical or personal procedures, and help define semi-private spaces in shared rooms. Privacy curtains can rapidly be contaminated within 1 week, yet their changing schedule is often infrequent [86]. Studies on drug-resistant *Acinetobacter baumannii* and *Streptococcus pyogenes* outbreaks in hospitals have identified curtains as a potential source of transmission [87,88]. Antimicrobial curtains with quaternary ammonium chlorides plus polyorganosiloxane and multilevel antimicrobial polymer (MAP-1) have been shown to effectively reduce the microbial burden and MDRO contamination compared with the standard curtain for an extended period in an active hospital setting [89,90]. Antimicrobial curtains provide an opportunity to avert indirect costs related to curtain changing and laundering in addition to improving patient safety. Their role in LTCFs is not adequately investigated.

### 4.10. Limitations

All seven included studies were conducted in the United States or Australia, which limits the generalizability of the findings, revealing a significant gap in evidence from the rest of the world. Moreover, LTCF design and management, financing, culture, resident density, climate, and prevalent pathogen profiles vary greatly across regions [47,91]. The effectiveness, cost–benefit ratio, and practical implementation barriers of methods and technologies for environmental disinfection may differ substantially across regions with different family structures and care models. This gap indicates a pressing need for context-specific research to inform appropriate and sustainable infection prevention strategies in LTCFs globally.

## 5. Conclusions

### Public Health Implications and Conclusive Remark

The future of healthcare environment hygiene is clear, as the field becomes increasingly recognized as a key component of successful infection prevention strategies. Still, more research is needed to prove the efficacy of standard interventions and of recently developed technologies [78].

Our scoping review showed that the landscape of environmental disinfection in LTCFs is moving toward no-touch technologies as adjuncts within multimodal IPC and cleaning bundles, comprising training, auditing, feedback, and improved workflows for manual methods. Bundled approaches have stronger evidence for reducing HAIs in healthcare settings than single components alone. In this regard, the policy relevance of our review is limited, as most studies report surrogate endpoints rather than infections, hospitalizations, or mortality. More studies with stronger study designs with clinical outcomes are required before implementation recommendations can be drawn. In addition, practical data on costs, staffing, time burden, maintenance, safety, and resident acceptability are required to guide public health decisions.

Key evidence gaps exist for LTCFs, where the unique challenges of their residential nature, vulnerable population, resources, and workforce constraints are central, and demand solutions distinct from those in acute care. Future success depends on generating stronger clinical evidence, developing integrated, practical bundles, and expanding research across diverse global contexts to protect the health of the growing elderly population in long-term care worldwide.

## Figures and Tables

**Table 1:** Summary of characteristics of included studies (*n* = 7).

Author	Country	Study Design	Setting	Disinfection Approach/ Intervention	Primary Clinical Infection Outcome (If Any)	Effect on Clinical Infection	Other Outcomes
Lesho 2022 [32]	USA	Observational with pre- and post-cleaning measurements	1 LTCF (3 units: dementia, rehabilitation, and residential)	Chlorine dioxide fogging ± wiping	None (SARS-CoV-2 RNA detection rate only)	N/A	67% surfaces showed SARS-CoV-2 RNA detection after fogging alone; −11% change with fogging plus wiping
Shoubridge 2023 [31]	Australia	Double-crossover, cluster RCT	4 LTCFs	Continuous UVGI appliance	Incidence rate of acute respiratory infections (ARIs)	No significant reduction per zone per cycle; modest reduction in total ARIs by study end	N/A
Cole 2023 [35]	USA	Pre–post study	1 LTCF	2 parts per billion of DHP in occupied setting	None (microbial CFU only)	N/A	Significant reduction in surface microbial load (controlling for location/area)
Cole 2025 [33]	USA	Observational outbreak report	1 LTCF (3 units)	<1 part per million DHP in occupied setting	COVID-19 attack rate	Attack rate: 66.67% (no DHP) vs. 0.00% (DHP), suggesting potential impact on airborne transmission	N/A
Jutkowitz 2023 [34]	USA	Single group interrupted time series	81 nursing homes	UV air purification system in HVAC system	COVID-19 cases or death	Weekly cases per 1000 residents: −1.69 (95% CI: −4.32, 0.95); probability of any case: −0.02 (95% CI: −0.04, 0.00), declines not statistically significant	N/A
Urrutia 2023 [36]	USA	Pre–post study	1 LTCF	AAPT—HVAC with UVGI	Healthcare-associated infection (HCAI) incidence	39.6% reduction in HCAIs	98.83% reduction in airborne pathogens; 89.88% reduction in VOCs
Sansom 2023 [37]	USA	Prospective multicenter observational study	6 ventilator-capable skilled nursing facilities	Hydrogen peroxide wipes	None (environmental contamination only)	N/A	*C. auris* contamination: 32.2% pre-disinfection vs. 20.5% post-disinfection

Abbreviations. LTCF: long-term care facility; RCT: randomized controlled trials; RNA: ribonucleic acid; UV: ultraviolet; UVGI: ultraviolet germicidal irradiation; ARI: acute respiratory infections; DHP: dry hydrogen peroxide; CFU: colony-forming unit; VOC: volatile organic compound; AAPT: advanced air purification technology; HVAC: heating, ventilation, and air conditioning; CI: confidence interval; N/A: not applicable.

**Table 2:** Pros and cons of novel disinfection approaches with emphasis on their applicability in LTCF settings.

Disinfection Method	Pros	Cons	Remarks
Chemical disinfection (HPV for terminal disinfection)	Demonstrates broad-spectrum and sporicidal efficacy, enabling significant reductions in environmental microbial load. Automated “no-touch” system standardizes decontamination and minimizes reliance on staff cleaning quality. Proven effectiveness in terminal disinfection following occupancy by infected or colonized individuals in hospital settings.	Requires dedicated equipment, room closure, and extended downtime for aeration, limiting throughput and flexibility in resident turnover. Implementation demands substantial capital investment, staff training, and ongoing maintenance. Efficacy is dependent on correct device operation, room configuration, and cycle validation. Potential safety and ventilation concerns for staff if protocols are not strictly followed.	HPV is a leading method for terminal disinfection in healthcare; however, evidence linking environmental decontamination to reduced infection rates in LTCFs remains limited and heterogeneous. Integration with routine cleaning and IPC protocols is essential for optimal outcomes.
Chemical disinfection (DHP in occupied setting)	Enables continuous disinfection while rooms are occupied, potentially reducing airborne and surface microbial contamination without requiring room vacancy. Automated delivery reduces human error and supports consistent application.	Efficacy may be lower compared to terminal high-concentration disinfection; dependent on device placement and air flow. Safety data in occupied spaces (especially for vulnerable populations) is still emerging; long-term effects require careful monitoring. Device maintenance and consumable costs add to operational burden.	DHP offers a practical option for ongoing environmental hygiene in LTCFs, particularly where frequent room closure is not feasible. Its role should be considered complementary to, not a replacement for, routine cleaning and targeted terminal disinfection.
Physical disinfection (UVGI for terminal disinfection)	Provides rapid, automated, broad-spectrum inactivation of pathogens on surfaces and in air. Reduces reliance on manual cleaning thoroughness and achieves high levels of microbial reduction in a standardized manner. Minimal chemical residues or odor.	Requires room vacancy during operation and protocols to avoid human exposure to UV-C. Effectiveness can be reduced by room geometry, shadowed areas, and surface irregularities. Equipment acquisition and maintenance represent additional costs.	UVGI is effective as a terminal disinfection modality in healthcare environments. Its operational success depends on integration with cleaning protocols and validation of effective coverage for all critical surfaces. Integration with routine cleaning and IPC protocols is essential for optimal outcomes.
Physical disinfection (shielded UVGI, visible light, far UV for continuous air purification)	Allow for ongoing or frequent disinfection in occupied spaces, contributing to sustained reduction in environmental bioburden. Can be used in the presence of staff and residents. Reduce risk of human error and supplement standard cleaning practices.	Efficacy can be variable and is often lower than terminal, unshielded UVGI; dependent on device placement and environmental factors. Initial capital costs and ongoing maintenance are required. Long-term safety and optimal dosing strategies, particularly for far-UV and shielded UV in vulnerable populations, are areas of active research.	Continuous disinfection technologies are a promising adjunct in LTCFs, particularly for high-traffic and high-touch areas. Robust clinical endpoint data are still limited, and integration with holistic IPC strategies is recommended.
Self-disinfecting coating	Provides passive, continuous antimicrobial activity on high-touch surfaces between cleaning cycles. Simple application to a variety of surfaces and materials; can reduce microbial persistence and cross-contamination risk.	Heterogeneous real-world efficacy; dependent on coating durability, maintenance, and compatibility with cleaning agents. Durability and reapplication: coatings may degrade with abrasion, cleaning agents, and time; reapplication costs and downtime must be planned. Compatibility concerns: potential interactions with cleaners, furniture, and equipment; risk of coating wear creating uneven protection or residues. Safety and environmental considerations: some formulations employ heavy metals or metal-oxide matrices; require evaluation of cytotoxicity, environmental release, and regulatory compliance.	Evidence for direct impact on infection rates in LTCFs remains limited; most studies focus on laboratory or surrogate endpoints. Self-disinfecting coatings can be a valuable component of a layered IPC approach but should not replace regular cleaning and disinfection. Selection of products with demonstrated durability and safety and periodic assessment of coating performance are key for effective implementation.

Abbreviations. HPV: hydrogen peroxide vapor; DHP: dry hydrogen peroxide; UVGI: ultraviolet germicidal irradiation; LTCF: long-term care facility; IPC: infection prevention and control measures.

**Table 3:** Key differences between acute hospital and long-term care (LTCF) settings and their implication in infection prevention and control measures.

Aspect	Hospital Setting	Long-Term Care Facility (LTCF)
Population/Residents	Acutely ill patients (wide age range); generally higher baseline health and cognitive function; most can understand and follow hygiene/isolation instructions.	Predominantly elderly, frail residents with multiple chronic conditions; very high rates of cognitive impairment (e.g., dementia) which hampers adherence to infection control measures.
Staffing and Training	Higher nurse-to-patient and staff-to-patient ratios; many staff with clinical training; dedicated infection prevention teams and regular IPC (infection prevention and control) training programs.	Lower staff-to-resident ratios; a larger proportion of non-clinical aides; few (or no) specialized infection prevention personnel. IPC training is often less standardized.
Cognitive/Behavioral	Patients typically cooperative and able to follow protocols (hand hygiene, isolation precautions) when instructed.	Many residents have dementia/frailty, making it difficult to maintain hand hygiene or follow isolation/distancing recommendations (e.g., wandering or participating in communal activities despite outbreaks).
Environment/Infrastructure	Predominantly private or semi-private rooms; acute-care wards with controlled ventilation and “hospital-grade” surfaces designed to be cleaned easily.	More home-like, communal layout—shared dining/recreation areas and often shared bathrooms. Indoor communal spaces (e.g., shared toilets, activity rooms) are common, which can facilitate pathogen transmission.
Infection Control Implementation	Rigorous, standardized IPC protocols guided by clear hospital policies (e.g., WHO/CDC guidelines). Hand hygiene, PPE use, and surveillance are strictly enforced and audited.	IPC measures are adapted from hospital protocols but are harder to enforce. LTCFs require tailored programs—basic hygiene (handwashing), PPE, staff education, and active surveillance—but resource limits and resident needs often impede full implementation.
Disinfection Practices	Routine manual cleaning using hospital-grade disinfectants (e.g., bleach, hydrogen peroxide formulations) with ≥99.9% pathogen killed; more widespread use of “no-touch” technologies (UV-C light, hydrogen peroxide vapor) for terminal cleaning.	Traditionally rely on manual cleaning/low-level disinfectants (quats, bleach wipes). Newer automated methods are being studied but evidence and experience remained limited.
Infection Risks and Outcomes	Well-documented hospital-acquired pathogen risk (e.g., MRSA, VRE, *C. difficile*) from contaminated surfaces; many studies link hospital cleaning protocols to reduced HAI rates.	Residents have high baseline rates of respiratory infections and MDRO colonization. Outbreaks often trace back to communal areas. Few LTCF studies exist; those available show environmental contamination reductions with new technologies, but evidence linking disinfection to fewer resident infections remains sparse.

## Data Availability

No new data were created or analyzed in this study.

## Supplementary Materials

The following supporting information can be downloaded at https://www.mdpi.com/article/10.3390/microorganisms14071408/s1. Table S1. Preferred Reporting Items for Systematic reviews and Meta-Analyses extension for Scoping Reviews (PRISMA-ScR) Checklist. Table S2. The full text of searching strategies. Figure S1. The PRISMA flow diagram.
