# Supplementary material for: Environmental Disinfection in Long-Term Care Facilities—A Scoping Review
Source: Microorganisms. 2026 Jun 26;14(7):1408. doi: 10.3390/microorganisms14071408 (PMC13414427; doi:10.3390/microorganisms14071408)

Supplementary materials

Environmental disinfection in long-term care facilities – A scoping review

Yinan He [1], Wing Sum Lo [2], Pak Leung Yuen [3], Patricia Tai Yin Ching [4], Eric Po Tung Sze [5], Kin On Kwok [6], argaret Ip [1] and Christopher Koon Chi Lai [1,*]

Table S1. Preferred Reporting Items for Systematic reviews and Meta-Analyses extension for Scoping Reviews (PRISMA-ScR) Checklist.

SECTION	ITEM	PRISMA-ScR CHECKLIST ITEM	REPORTED ON PAGE #
TITLE			
Title	1	Identify the report as a scoping review.	1
ABSTRACT			
Structured summary	2	Provide a structured summary that includes (as applicable): background, objectives, eligibility criteria, sources of evidence, charting methods, results, and conclusions that relate to the review questions and objectives.	1-2
INTRODUCTION			
Rationale	3	Describe the rationale for the review in the context of what is already known. Explain why the review questions/objectives lend themselves to a scoping review approach.	2
Objectives	4	Provide an explicit statement of the questions and objectives being addressed with reference to their key elements (e.g., population or participants, concepts, and context) or other relevant key elements used to conceptualize the review questions and/or objectives.	3
METHODS			
Protocol and registration	5	Indicate whether a review protocol exists; state if and where it can be accessed (e.g., a Web address); and if available, provide registration information, including the registration number.	3
Eligibility criteria	6	Specify characteristics of the sources of evidence used as eligibility criteria (e.g.,	3

SECTION	ITEM	PRISMA-ScR CHECKLIST ITEM	REPORTED ON PAGE #
		years considered, language, and publication status), and provide a rationale.	
Information sources*	7	Describe all information sources in the search (e.g., databases with dates of coverage and contact with authors to identify additional sources), as well as the date the most recent search was executed.	3
Search	8	Present the full electronic search strategy for at least 1 database, including any limits used, such that it could be repeated.	4, Supplementary materials p4
Selection of sources of evidence†	9	State the process for selecting sources of evidence (i.e., screening and eligibility) included in the scoping review.	4
Data charting process‡	10	Describe the methods of charting data from the included sources of evidence (e.g., calibrated forms or forms that have been tested by the team before their use, and whether data charting was done independently or in duplicate) and any processes for obtaining and confirming data from investigators.	4
Data items	11	List and define all variables for which data were sought and any assumptions and simplifications made.	4
Critical appraisal of individual sources of evidence§	12	If done, provide a rationale for conducting a critical appraisal of included sources of evidence; describe the methods used and how this information was used in any data synthesis (if appropriate).	N/A
Synthesis of results	13	Describe the methods of handling and summarizing the data that were charted.	4
RESULTS			
Selection of sources of evidence	14	Give numbers of sources of evidence screened, assessed for eligibility, and included in the review, with reasons for exclusions at each stage, ideally using a flow diagram.	4

SECTION	ITEM	PRISMA-ScR CHECKLIST ITEM	REPORTED ON PAGE #
Characteristics of sources of evidence	15	For each source of evidence, present characteristics for which data were charted and provide the citations.	5-6
Critical appraisal within sources of evidence	16	If done, present data on critical appraisal of included sources of evidence (see item 12).	N/A
Results of individual sources of evidence	17	For each included source of evidence, present the relevant data that were charted that relate to the review questions and objectives.	5-6
Synthesis of results	18	Summarize and/or present the charting results as they relate to the review questions and objectives.	6-9
DISCUSSION			
Summary of evidence	19	Summarize the main results (including an overview of concepts, themes, and types of evidence available), link to the review questions and objectives, and consider the relevance to key groups.	9-23
Limitations	20	Discuss the limitations of the scoping review process.	23
Conclusions	21	Provide a general interpretation of the results with respect to the review questions and objectives, as well as potential implications and/or next steps.	23-24
FUNDING			
Funding	22	Describe sources of funding for the included sources of evidence, as well as sources of funding for the scoping review. Describe the role of the funders of the scoping review.	N/A

Table S2. The full text of searching strategies.

Database	Searching strategy
**PubMed**	("environmental disinfection" OR "environmental contamination" OR "Disinfection"[MeSH] OR "no-touch disinfection"[tiab] OR "automated room disinfection"[tiab] OR "robotic disinfection"[tiab] OR "electrostatic spray"[tiab] OR "Ultraviolet Rays"[MeSH] OR "ultraviolet light"[tiab] OR "UVC"[tiab] OR "UV-C"[tiab] OR "ultraviolet germicidal irradiation"[tiab] OR "UVGI"[tiab] OR "ultraviolet" OR "Surface Coatings"[MeSH] OR "Antimicrobial Surfaces"[MeSH] OR "Self- disinfecting surface" OR "cationic coating"[tiab] OR "cationic polymer"[tiab] OR "quaternary ammonium coating"[tiab] OR "polymeric antimicrobial surface"[tiab] OR "C-Polar"[tiab] OR "C Polar"[tiab] OR "Heat ventilation air" OR "ventilation air" OR "Hydrogen peroxide[tiab]" OR "Alcohol[tiab]" OR "Sodium hypochlorite" OR "Glutaraldehyde" OR "Chemical Disinfection" OR "Ortho phthal aldehyde" OR "iodine" OR "Disinfectants" OR "Triclosan" OR "heavy metal" OR "Copper coated" OR "Light activated antimicrobials" OR "Titanium dioxide") AND ("old age home" OR "OAH" OR "Homes for the Aged"OR "Nursing Homes" OR "Residential Facilities" OR "Long-Term Care" OR "Assisted Living Facilities" OR "old age home" OR "elderly care" OR "elder care" OR "residential care" OR "nursing home" OR "long term care" OR "healthcare facilities" OR "assisted living" OR "skilled nursing" OR "residential care facilities") AND ("2021/01/01"[Date - Publication] : "2026/05/02"[Date - Publication])
**Medline**	1. (exp Disinfection/ or exp Sterilization/ or (disinfect* or steriliz* or decontamination).mp. or ((environment* or surface*) adj3 (disinfect* or clean* or decontaminat*)).mp.) and (exp Nursing Homes/ or exp Homes for the Aged/ or exp Assisted Living Facilities/ or (nursing home* or "old age home*" or "aged care" or "long term care" or “skilled nursing” or “residential aged care” or “geriatric care facilities”).mp.)<br>(N=387)<br><br>2. limit 1 to yr="2021 – current(2026/05/02)"
**Embase**	1. (exp Disinfection/ or exp Sterilization/ or (disinfect* or steriliz* or decontamination).mp. or ((environment* or surface*) adj3 (disinfect* or clean* or decontaminat*)).mp.) and (exp Nursing Homes/ or exp Homes for the Aged/ or exp Assisted Living Facilities/ or (nursing home* or "old age home*" or "aged care" or "long term care" or “healthcare facilities” or"skilled nursing" or "residential aged care" or "geriatric care facilities").mp.)<br>2. limit 1 to ("remove medline records" and yr="2021 – current (2026/05/02)")
**CINAHL**	1. ("environmental disinfection" OR "environmental contamination" OR "Disinfection" OR "no-touch disinfection" OR "automated room

	disinfection" OR "robotic disinfection" OR "electrostatic spray" OR "Ultraviolet Rays" OR "ultraviolet light" OR "UVC" OR "UV-C" OR "ultraviolet germicidal irradiation" OR "UVGI" OR "ultraviolet" OR "Surface Coatings" OR "Antimicrobial Surfaces" OR "Self- disinfecting surface" OR "cationic coating" OR "cationic polymer" OR "quaternary ammonium coating" OR "polymeric antimicrobial surface" OR "C-Polar" OR "C Polar" OR "Heat ventilation air" OR "ventilation air" OR "Hydrogen peroxide" OR "Sodium hypochlorite" OR "Glutaraldehyde" OR "Chemical Disinfection" OR "Ortho phthal aldehyde" OR "iodine" OR "Disinfectants" OR "Triclosan" OR "heavy metal" OR "Copper coated" OR "Light activated antimicrobials" OR "Titanium dioxide")) AND (MH "Long Term Care" OR MH "Long Term Care Nurses" OR MH "Long Term Care Nursing" OR MH "Alternative Health Facilities" OR "old age home" OR "OAH" OR "Homes for the Aged"OR "Nursing Homes" OR "Residential Facilities" OR "Long-Term Care" OR "Assisted Living Facilities" OR "old age home" OR "elderly care" OR "elder care" OR "residential care" OR "nursing home" OR "long term care" OR "healthcare facilities" OR "assisted living" OR "skilled nursing" OR "residential care facilities")<br>2. filter: 01/2021 – 05/2026
**Scopus**	("environmental disinfection" OR "environmental contamination" OR "Disinfection" OR "no-touch disinfection" OR "automated room disinfection" OR "robotic disinfection" OR "electrostatic spray" OR "Ultraviolet Rays" OR "ultraviolet light" OR "UVC" OR "UV-C" OR "ultraviolet germicidal irradiation" OR "UVGI" OR "ultraviolet" OR "Surface Coatings" OR "Antimicrobial Surfaces" OR "Self- disinfecting surface" OR "cationic coating" OR "cationic polymer" OR "quaternary ammonium coating" OR "polymeric antimicrobial surface" OR "C-Polar" OR "C Polar" OR "Heat ventilation air" OR "ventilation air" OR "Hydrogen peroxide" OR "Sodium hypochlorite" OR "Glutaraldehyde" OR "Chemical Disinfection" OR "Ortho phthal aldehyde" OR "iodine" OR "Disinfectants" OR "Triclosan" OR "heavy metal" OR "Copper coated" OR "Light activated antimicrobials" OR "Titanium dioxide") AND ("old age home" OR "OAH" OR "Homes for the Aged"OR "Nursing Homes" OR "Residential Facilities" OR "Long-Term Care" OR "Assisted Living Facilities" OR "old age home" OR "elderly care" OR "elder care" OR "residential care" OR "nursing home" OR "long term care" OR "healthcare facilities" OR "assisted living" OR "skilled nursing" OR "residential care facilities") AND NOT (“review”)

Figure S1. The PRISMA flow diagram.

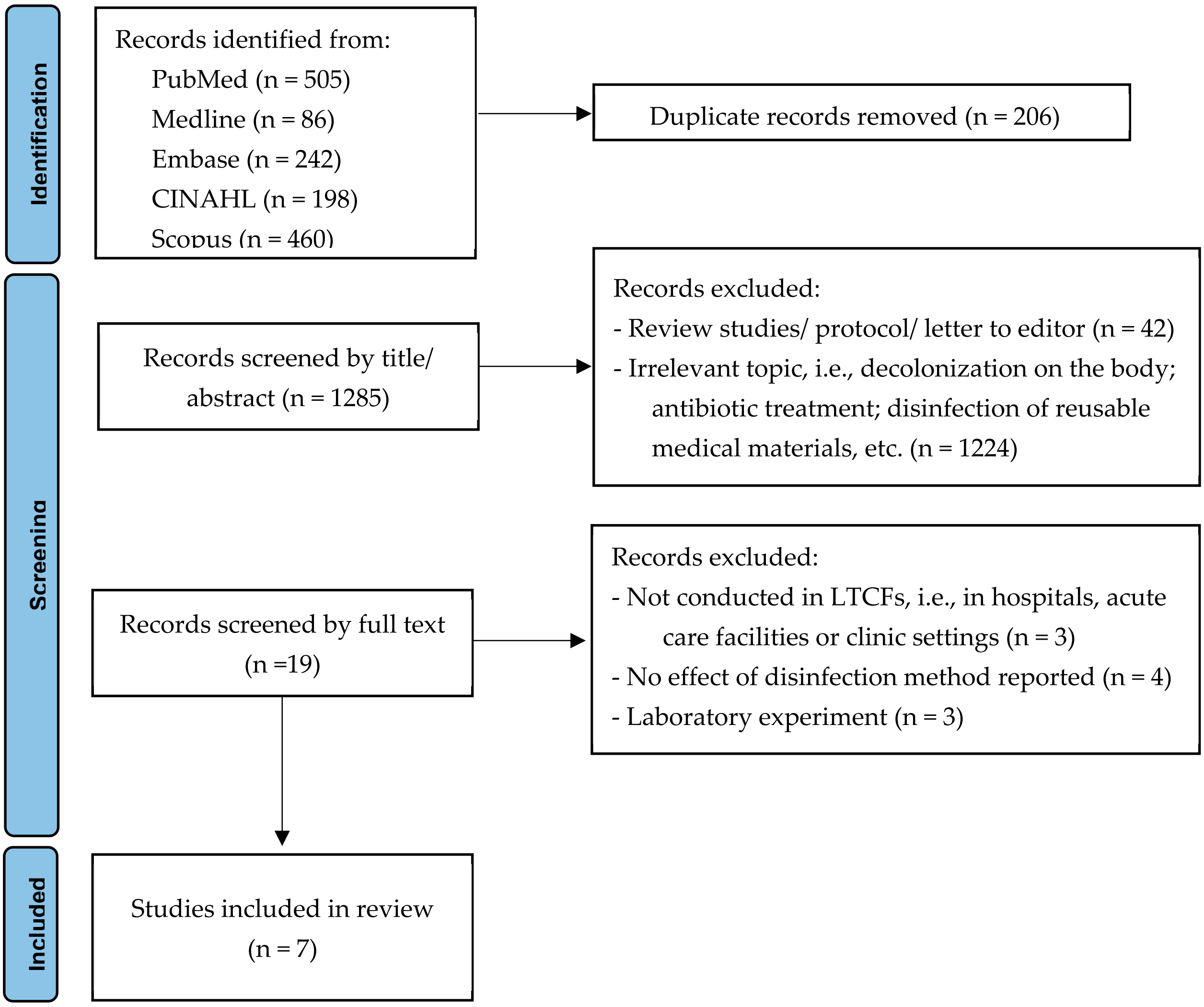

Supplement: Supplementary file 1 [file microorganisms-14-01408-s001.zip › microorganisms-4285484-supplementary.pdf]
